# Reply to: Congenital nystagmus, disability, visual impairment, and noncompaction suggest hereditary disease

**DOI:** 10.31744/einstein_journal/2019CE5401

**Published:** 2019-11-13

**Authors:** Ednei Costa Maia, Filippo Aragão Savioli, Sanna Roque Pinheiro, Leandro Santini Echenique, Japy Angelini Oliveira

**Affiliations:** 1 Universidade Federal de São Paulo, São Paulo, SP, Brazil.; 2 Hospital Israelita Albert Einstein, São Paulo, SP, Brazil.

Thank you for interest in our paper.

The manuscript was written to discuss the question about sports eligibility in Paralympic athletes. Our case did not show familiar antecedents of cardiac disease. During the study, we could not have access to patient’s family history to verify her family incidence of cardiopathy.

