# Supplementary material for: Reply to: Congenital nystagmus, disability, visual impairment, and noncompaction suggest hereditary disease
Source: Einstein (Sao Paulo). 2019 Nov 13;17(4):eCE5401. doi: 10.31744/einstein_journal/2019CE5401 (PMC6855695; doi:10.31744/einstein_journal/2019CE5401)
Supplement: Translation

# Translation: Resposta para: Nistagmo congênito, deficiência, disfunção visual e não compactação sugerem doença hereditária

**Authors:** Ednei Costa Maia, Filippo Aragão Savioli, Sanna Roque Pinheiro, Leandro Santini Echenique, Japy Angelini Oliveira

**Affiliations:** 1 Universidade Federal de São Paulo, São Paulo, SP, Brasil.; 2 Hospital Israelita Albert Einstein, São Paulo, SP, Brasil.

Agradecemos o interesse em nosso estudo.

O artigo foi escrito para discutir a questão sobre elegibilidade esportiva em atletas paraolímpicos. Em nosso caso, o paciente não demostrou histórico familiar de doença cardíaca. Em nosso estudo, não foi possível ter acesso ao histórico da família do paciente para checar a incidência de cardiopatia.
